# P-1205. *In vitro* activity of EL219 (SF001) and amphotericin B agents against a collection of *Candida* and *Aspergillus* isolates collected in 2024

**DOI:** 10.1093/ofid/ofaf695.1398

**Published:** 2026-01-11

**Authors:** S J Ryan Arends, Paul Rhomberg, Mariana Castanheira

**Affiliations:** Element Iowa City (JMI Laboratories), North Liberty, IA; Element Materials Technology/Jones Microbiology Institute, North Liberty, Iowa; Element, North Liberty, IA

## Abstract

**Background:**

EL219 is a novel polyene antifungal drug (formerly known as SF001) currently in preparation for Phase 2 clinical trials (Elion Therapeutics). Through synthetic modifications to amphotericin B, EL219 was designed to improve antifungal activity while reducing the potential for toxicity. This international surveillance study reports on the *in vitro* activity of EL219 and amphotericin B tested against contemporary *Candida* and *Aspergillus* clinical isolates.

**Figure:**
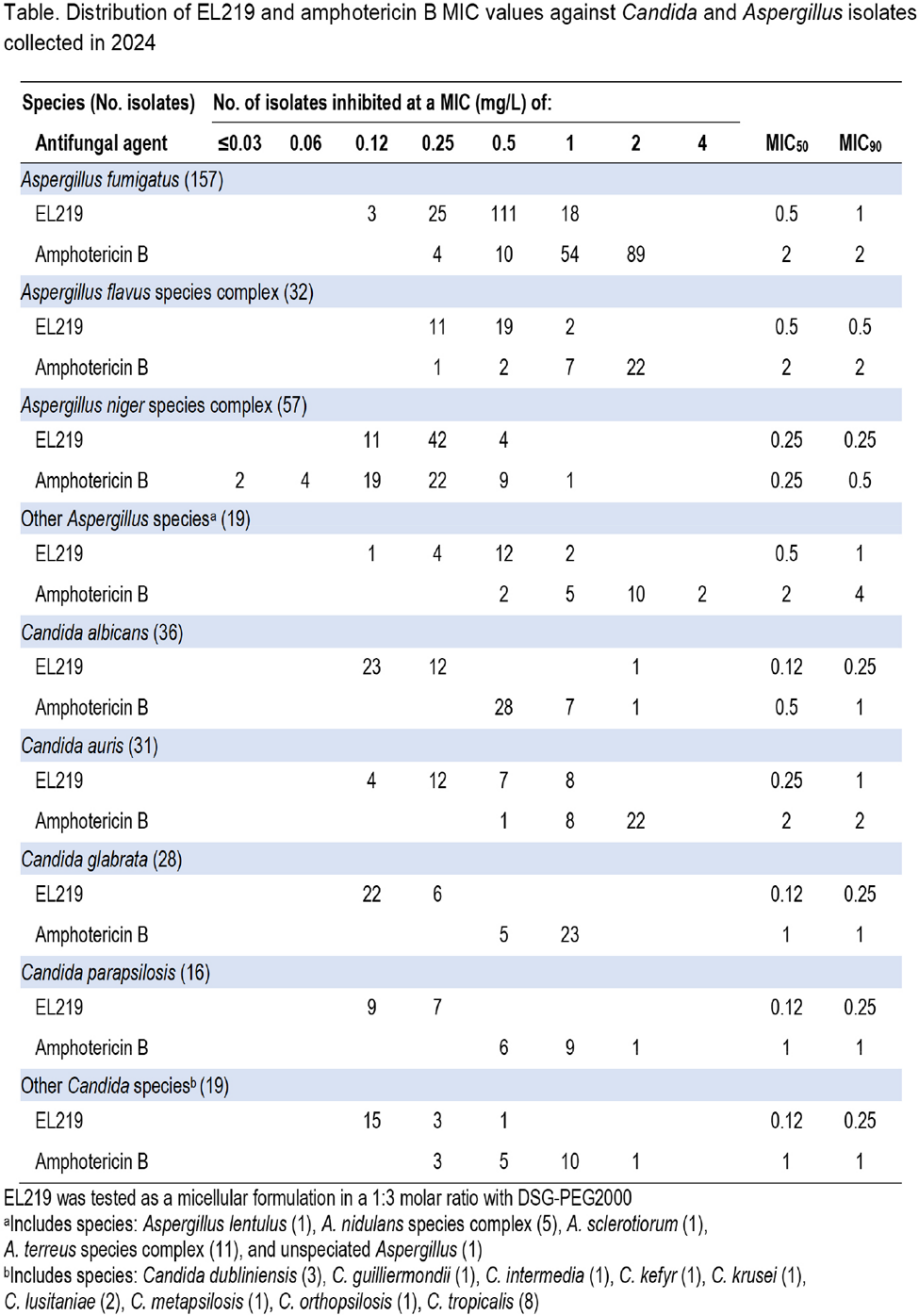

**Methods:**

130 *Candida* and 265 *Aspergillus* isolates were collected in 2024 from 73 medical centers worldwide including: USA (n=118, 25 centers), Europe (n=190, 17 countries), Asia-Pacific (n=75, 6 countries), and Latin America (n=12, 5 countries). Isolates were tested for susceptibility by CLSI broth microdilution methods. MIC results for comparator agents were interpreted per CLSI guidelines.

**Results:**

EL219 was active against all *Aspergillus* species with MIC_90_ values ranging from 0.25-1 µg/mL; *A. fumigatus* (n=157; MIC_50/90_, 0.5/1 µg/mL), *A. flavus* species complex (sc) (n=32; MIC_50/90_, 0.5/0.5 µg/mL), *A. niger* sc (n=57; MIC_50/90_, 0.25/0.25 µg/mL), other *Aspergillus* species (n=19; MIC_50/90_, 0.5/1 µg/mL). EL219 was more active *in vitro* than amphotericin B against all *Aspergillus* species groups whose MIC_90_ values ranged from 0.5-4 µg/mL. EL219 was also active against all *Candida* species with MIC_90_ values ranging from 0.25-1 µg/mL; *C. albicans* (n=36; MIC_50/90_, 0.12/0.25 µg/mL), *C. auris* (n=31; MIC_50/90_, 0.25/1 µg/mL), *C. glabrata* (n=28; MIC_50/90_, 0.12/0.25 µg/mL), *C. parapsilosis* (n=16; MIC_50/90_, 0.12/0.25 µg/mL), and other *Candida* species (n=19; MIC_50/90_, 0.12/0.25 µg/mL). EL219 was more active *in vitro* than amphotericin B against all *Candida* species groups whose MIC_90_ values ranged from 1-2 µg/mL.

**Conclusion:**

EL219 was active against prevalent *Aspergillus* and *Candida* clinical isolates causing invasive mycoses recovered in 2024. EL219 was often 4- to 8-fold more active than amphotericin B when comparing MIC_50/90_ values. These results further support the continued development of EL219.

**Disclosures:**

Mariana Castanheira, PhD, Melinta Therapeutics: Advisor/Consultant|Melinta Therapeutics: Grant/Research Support

